# HaKom – the Halle continuum as a postgraduate medical training curriculum, illustrated using the development and delivery of the ready for duty course

**DOI:** 10.3205/zma001714

**Published:** 2024-11-15

**Authors:** Susanna Jaspers, Lena Bauer, Linn Hempel, Juliane Achenbach, Josefin Bosch, Christiane Ludwig, Miriam Schwardt, Benjamin Reufsteck, Anna Siol, Jonas Steglich, Dietrich Stoevesandt

**Affiliations:** 1 University of Halle-Wittenberg, Medical Faculty, Dorothea Erxleben Learning Centre, Halle (Saale), Germany

**Keywords:** postgraduate training curriculum, postgraduate medical training, simulations

## Abstract

**Objective:**

The Halle continuum (HaKom) is a course for doctors undergoing postgraduate medical training in southern Saxony-Anhalt. It revises the skills and knowledge acquired during undergraduate medical training and develops them on an individual and needs-driven basis. The development and establishment of the three-day ready for (resident on-call) duty (*Fit für den Dienst*) course, which has been held four times a year since 2020, will be used as an example to present the overall HaKom curriculum.

**Methodology:**

The HaKom comprises a total of eight courses already established and four further courses still being planned, the order of which can be adapted individually. One of the HaKom courses is the ready for duty course, the structure and development of which will be explained below. The course content was developed in line with the KERN model using a questionnaire-based needs assessment conducted between 2019 and 2020 among 77 participants from ten different specialty areas.

**Results:**

In the in-person ready for duty course, 16 participants learn relevant content for their first (on-call) duty using simulations, communication training and practical skills stations at the learning centre of Halle University Hospital. Participants can study theoretical content in detail in a blended-learning approach. Evaluations of the curriculum taught to the first 12 cohorts – comprising a total of 205 participants – were conducted with a response rate of 65% and the course content was adapted on the basis of these evaluations. All of the respondents (100%) said that the course was useful in preparing them at the beginning of their medical career.

**Conclusion:**

As part of the overall HaKom curriculum, the ready for duty course builds on and goes beyond undergraduate medical training. By improving the quality of postgraduate medical training, it is designed to guarantee adequate patient care and can also promote local networking among the participants.

## Zusammenfassung

**Zielsetzung:**

Das Hallenser Kontinuum (HaKom) ist ein Kursangebot für Ärztinnen und Ärzte in Weiterbildung im südlichen Sachsen-Anhalt. Es wiederholt die im Studium erlernten Fertigkeiten und Kenntnisse und baut diese individuell und bedarfsgerecht aus. Anhand der Entwicklung und Etablierung des dreitägigen, seit 2020 viermal jährlich stattfindenden Kurses „Fit für den Dienst“ wird das Gesamtcurriculum HaKom beispielhaft vorgestellt.

**Methodik:**

Insgesamt umfasst das HaKom acht bereits etablierte sowie vier geplante Kurse, die in individueller Reihenfolge absolviert werden können. Teil des HaKom ist der „Fit für den Dienst“-Kurs, dessen Aufbau und Entwicklung im Folgenden erläutert werden soll. Die Kursinhalte wurden entsprechend dem KERN-Zyklus anhand einer von 2019 bis 2020 durchgeführten Fragebogenbedarfsanalyse mit 77 Teilnehmenden aus 10 Fachrichtungen entwickelt.

**Ergebnisse:**

Im „Fit für den Dienst“-Präsenzkurs werden 16 Teilnehmenden relevante Inhalte für den ersten (Bereitschafts)Dienst mittels Simulationen, Kommunikationstrainings sowie praktischen Skills-Stationen am Lernzentrum der Universitätsmedizin Halle vermittelt. Theoretische Inhalte können in einem „blended-learning“-Konzept vertieft werden. Die ersten 12 Durchgänge mit insgesamt 205 Teilnehmenden wurden mit einer Rücklaufquote von 65% evaluiert und die Kursinhalte entsprechend der Evaluationen angepasst. Für alle (100%) der Befragten stellte der Kurs ein sinnvolles Angebot für ihren Berufseinstieg dar.

**Fazit:**

Der Kurs „Fit für den Dienst“ stellt im Gesamtcurriculum HaKom eine Weiterführung der medizinischen Ausbildung über das Medizinstudium hinaus dar. Er soll durch eine Qualitätsverbesserung der Weiterbildung die Patientenversorgung sicherstellen und kann zudem eine lokale Vernetzung der Teilnehmenden bewirken.

## 1. Introduction

The aim of postgraduate medical training is for doctors to learn, develop and apply medical skills to ensure the quality of medical care provided in the interests of patients [1], [2].

In the German-speaking countries, the undergraduate medical training curriculum sets out cross-curricular study units that build on each other, with theoretical, practical and communication skills gradually becoming more advanced (German Licensing Regulations for Physicians (in German): [https://www.gesetze-im-internet.de/_appro_2002/BJNR240500002.html]); these units are extended and standardised in the National Competence Based Catalogue of Learning Objectives for Undergraduate Medical Education (NKLM) [3]. In contrast, in postgraduate medical training, skills are primarily taught specifically for the particular specialty and position [4], [5], [6], [7]. The amount of subject matter taught during medical training is steadily increasingly and the necessary expertise is becoming more and more complex [8], [9]. A large number of postgraduate medical training and continuous medical education (CME) courses are available. Cross-curricular medical training content that can be accessed individually is also provided online, for example in online knowledge libraries, or through conventional media, such as textbooks [8], [10].

The aim of the Halle continuum (HaKom) is to establish a structured postgraduate medical training concept coordinated within the individual parts of the course for the period between obtaining a licence to practise and taking the specialty examination. The necessary skills are to be taught across the various areas of specialisation, adapted to each doctor’s individual postgraduate medical training pathway and tailored to the conditions that prevail where the doctor is training, offering the opportunity to apply knowledge in a safe environment. This is designed to guarantee a quality standard in the treatment of patients. As part of the HaKom, the ready for duty (*Fit für den Dienst*) course will be described below as an example to illustrate the development of the entire HaKom curriculum.

## 2. Project description

### 2.1. HaKom (Halle continuum)

The HaKom is designed to teach subject matter that is fundamental to a doctor’s work, regardless of the particular area of specialisation. The diverse courses developed for this purpose are not planned in a strictly linear manner, but instead can be completed as required in line with the individual postgraduate medical training pathway. The skills learnt during undergraduate medical training are to be revised, at the same time adding up-to-date knowledge relevant to the profession. The target group varies depending on the course and medical discipline involved. The courses are geared towards junior doctors (medical school graduates undergoing postgraduate medical training) from all the different areas of specialisation with departments with patient beds in the region. The doctors often begin with the ready for duty course, followed by further HaKom courses adapted to their individual training pathway. The courses are CME accredited and the participants are given time off by their hospital or department to attend the courses. Depending on the course, an invitation is usually sent directly to the head of the relevant hospital or department or the course may be advertised publicly.

The HaKom is coordinated by the Dorothea Erxleben Learning Centre (DELH) at the Faculty of Medicine of the University of Halle-Wittenberg. With a few exceptions, it is held at the DELH itself and is run in cooperation with the departments and hospitals of Halle University Hospital and with postgraduate training hospitals in the region.

The coordinators’ positions are financed jointly by the two institutions and the courses are partly refinanced. Depending on the course and the associated competencies required, lecturers come from Halle University Medicine or from the relevant medical disciplines at the nearby hospitals in southern Saxony-Anhalt. In addition, some of the topics are supervised by students with relevant training as tutors (for example the sonography courses). Some of the courses are refinanced through course fees in cooperation with the Medical Association of the State of Saxony-Anhalt.

In addition to courses that have been an established part of the curriculum for some time now, such as the emergency medicine course defined in the Model Specialty Training Regulations (*Musterweiterbildungsordnung*) adopted by the German Medical Association (*Bundesärztekammer*), there are also newer formats, such as the digitalisation curriculum (Digitalisation in Medicine – Postgraduate Medical Training Curriculum, CÄWIN) and the Ready to Teach course. Some courses also contain interprofessional content and include participants from the field of nursing or paramedics.

Like the ready for duty course described below, the HaKom events were developed on the basis of the Kern approach [11] and are regularly evaluated (e.g. CÄWIN course).

**Figure 1:**
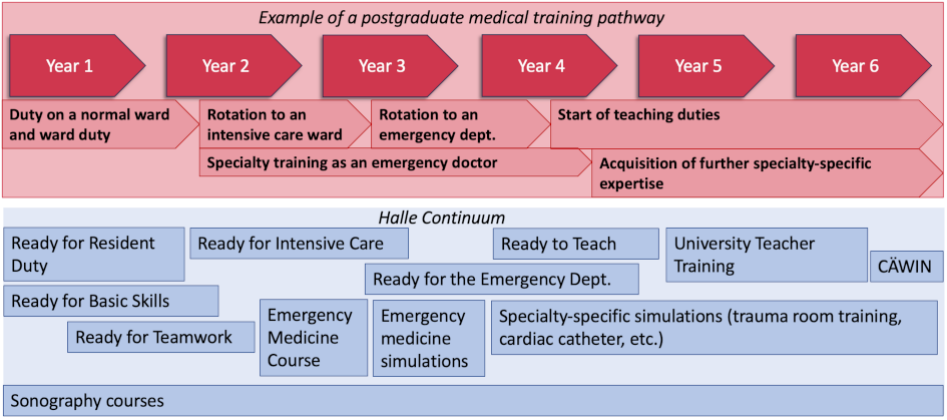
Example of a postgraduate medical training pathway (red) parallel to the Halle continuum (HaKom) (blue) coordinated to fit in with the postgraduate medical training and rotation pathway

The following courses are currently offered (see also figure 1 (Fig. 1)):

- *ready for duty (3 days)*: teaching and revising competencies to master the challenges that may occur during the first few times on duty/ward duty
- *ready to teach (2 days):* training to acquire teaching skills for medical and research professionals at the start of their career
- *ready for intensive care (1 day)*: teaching necessary competencies before doctors work on an intensive care ward
- *CÄWIN (blended learning)*: acquiring competencies relating to digitalisation in medicine [12]
- *university teacher training (6 days)*: acquiring competencies in medical didactics und curricular teaching
- *specialty-specific simulation*: traumatology- and non-traumatology-based trauma room training in hospital, cardiac catheter simulation
- *emergency medicine course* based on the German medical association’s model specialty training regulations
- *sonography* courses

The following course formats are currently being devised and finalised:

- *ready for the emergency department (3 days)*: teaching competencies to work as a doctor in an emergency department
- *ready for teamwork (2 days)*: teaching and reflecting on team and leadership skills
- *ready for basic skills (2 days)*: revising and expanding on practical skills already taught as part of undergraduate medical training
- *NASim (3 days)*: emergency medicine simulations

### 2.2. The ready for duty course

**Figure 2:**
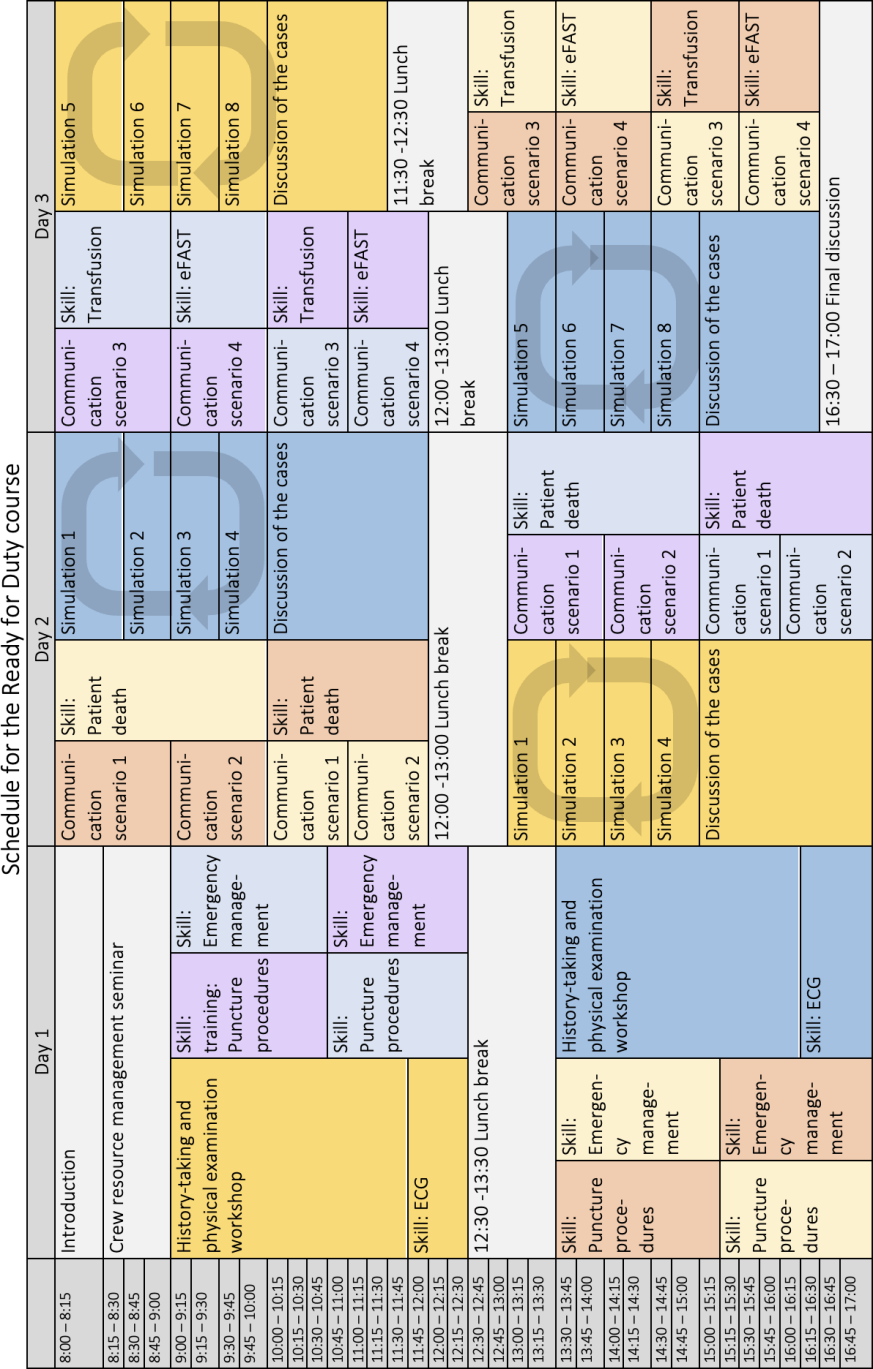
Ready for Duty blueprint developed on the basis of a needs analysis. A maximum of 16 participants are divided into four groups of four people each, shown here in different colours (light yellow, orange, light purple, light blue). Some of the units take place in larger groups of eight participants (dark yellow, blue). In the simulations, the participants rotate between the four scenarios, marked by the arrows. Abbreviations: ECG: electrocardiography; eFAST: extended Focused Assessment with Sonography for Trauma

As part of the HaKom, the ready for duty course was developed to prepare junior doctors for the potential challenges of regular and on-call duty. The three-day course is therefore primarily geared towards doctors at the beginning of their postgraduate medical training and before their first on-call duty. Most of the participants work at Halle University Hospital, although some work at other hospitals in the region. The ready for duty course is designed for all departments with patient beds and is a CME-accredited course worth 30 credits. The course content was developed in line with the Kern approach to curriculum development in medical education [11] based on a needs analysis conducted between the end of 2019 and the beginning of 2020. To begin with, the target group was defined: doctors at the start of their medical career who face similar medical emergencies and problems from different areas of specialisation. At the beginning of the process of developing the course, focus group interviews were carried out with the departments to which the course might be relevant with a view to exploring the need for this type of course and its potential content. Specifically, standardised emails were sent to the secretary’s office of the relevant departments (the internal medicine departments, the central emergency department, the surgical departments, the anaesthesiology and neurology departments of Halle University Hospital) to ask about doctors at the beginning of their medical career and teaching staff, and these hospital staff members were subsequently contacted. Based on the responses recorded in these 35 focus group interviews, an extensive online questionnaire containing 118 items was developed and sent to all departments with patient beds that use a duty rota system and to the central emergency department. The questionnaire was completed by junior doctors (n=58) and by specialists, consultants, and senior consultants (n=20) from ten different areas of specialisation (including internal medicine, neurology, anaesthesiology and surgery). The questions in the survey concerned the relevance of 35 practical skills, 24 communication topics, 15 theoretical topics, 16 clinical treatment scenarios, 11 questions about hospital organisation, four items about mental hygiene and 13 items about the general course organisation; the responses were used to identify potential course content. After the general conditions had been established, the learning objectives and learning forms were drawn up for the individual modules, thus creating the course programme explained below (see figure 2 (Fig. 2)).

## 3. Results

The course lasts for three days (=15 study units) and is designed for 16 participants each time. In order to cover as much subject matter as possible and to reduce the amount of “downtime” for the lecturers, the course was planned such that the 16 participants are divided up into four groups, each of which undergo the parts of the programme at a different time and in a different order from the other groups (see figure 2 (Fig. 2)).

Core elements of the course include applying theoretical content in practice, practising skills and trying out tasks in a safe environment. Lecture formats are reduced to a minimum. The only one is the talk on crew resource management (1 study unit). To teach the study objectives, the curriculum uses simulations, case-based learning (emergency management, postmortem examinations, death certificates, transfusion, history-taking and physical examinations, ECG), practical stations with feedback (extended Focused Assessment with Sonography in Trauma – eFAST, puncture procedures) and virtual reality technology (postmortem examinations). The flipped classroom approach is used to ensure that participants begin the course with the same level of knowledge, with study content being provided prior to the start of the course. This means that theoretical content can be revised individually in a blended-learning approach, freeing up the lesson time for practice and application [13], [14]. This online study content includes areas such as emergency management, the structure of the duty system at Halle University Hospital, ECG assessment, important content on anticoagulation treatment, drawing up post-consultation letters and operation reports, basic aspects of pain medicine, and the procedure to be followed in the event of patient elopement or after patient death.

Simulations, in which participants are confronted with typical scenarios from clinical practice, are one of the key elements of the course. Simulation actors with the relevant training [15] replicate situations such as acute dyspnoea, abdominal pain or an inpatient fall. The course participants take part in a total of eight simulations in tandems over two days and have 30 minutes for each simulation. After taking the patient’s history and performing a physical examination (for which findings are provided in writing in some cases), participants have to combine the results of the examination with information from a patient’s file and then set out a suspected diagnosis and treatment options. What is special about this unit is that the overall organisation of a hospital ward can be simulated and used: the tandems are supposed to consult a backup service for advice and to call on nursing staff (played by student assistants) for specific assistance on the case (drawing up medication, obtaining materials, attaching monitors, etc.). The simulation therefore not only focuses on the medical aspects of the cases but also helps the participants practise efficient, respectful communication between professionals and with the patients. After participants have completed all the simulations, there is a round of joint evaluation and reflection supervised and moderated by experienced medical colleagues, during which the cases are discussed in terms of aspects and insights regarding specialist medical topics, team communication and organisation, thus consolidating the learning content. The simulation scripts were reviewed by specialists after they had been produced.

There are several units involving encounters with simulated patients in the ready for duty course designed to explicitly train communication skills. The priority topics build on the basic knowledge and complexity from undergraduate medical training in order to avoid redundancies while at the same time revising study content. They include dealing with treatment errors within a team, dealing with demanding and angry relatives, and having difficult telephone conversations. In addition, a course has been established on talking professionally about dying, focusing on personal discussions between colleagues on the topic. This is rounded off by video content from the DocCom.deutsch module [16], which provides help and tips for this sometimes very difficult and stressful type of conversation.

The modules provide an opportunity to practise the skills that they focus on: emergency sonography, transfusing blood products, symptom-based history-taking and physical examinations, ECG evaluation and management of medical emergencies. In addition, participants can use different models to revise various puncture procedures (for example lumbar puncture or inserting a central venous catheter).

Developing and running a course requires extensive preparation and ongoing updating of various content. After the number and set-up of the rooms had been decided on (for example camera transmission at the communication stations), lists of materials were drawn up, including blood products for the transfusion station, simulators for the emergency management station, a doll for the postmortem examination station, models for inserting central venous catheters or arterial catheters, monitoring and medication for the simulation scenarios and an ultrasound machine.

Lecturers (for the history-taking and physical examination station, emergency management, postmortem examinations and death certificates, etc.) are recruited from Halle University Medicine and include both specialists and experienced junior doctors. Some parts of the programme are taught by specialised student tutors who have undergone the relevant training (including written preparatory materials); student tutors mainly teach participants at the eFAST station but sometimes they may also cover the puncture station. Simulated patients are an important part of staff planning for the course. They are chosen based on the simulation cases (age and sex are often the decisive criteria) and the scripts for these roles are provided in advanced; they also take part in regular training sessions. On the day of the course, the relevant make-up for the role (cyanosis, pallor, icterus, head laceration, etc.) is applied to the simulated patients and they are given the relevant medical materials and equipment (venous catheters with infusions, monitoring) by specialised simulation staff.

**Figure 3:**
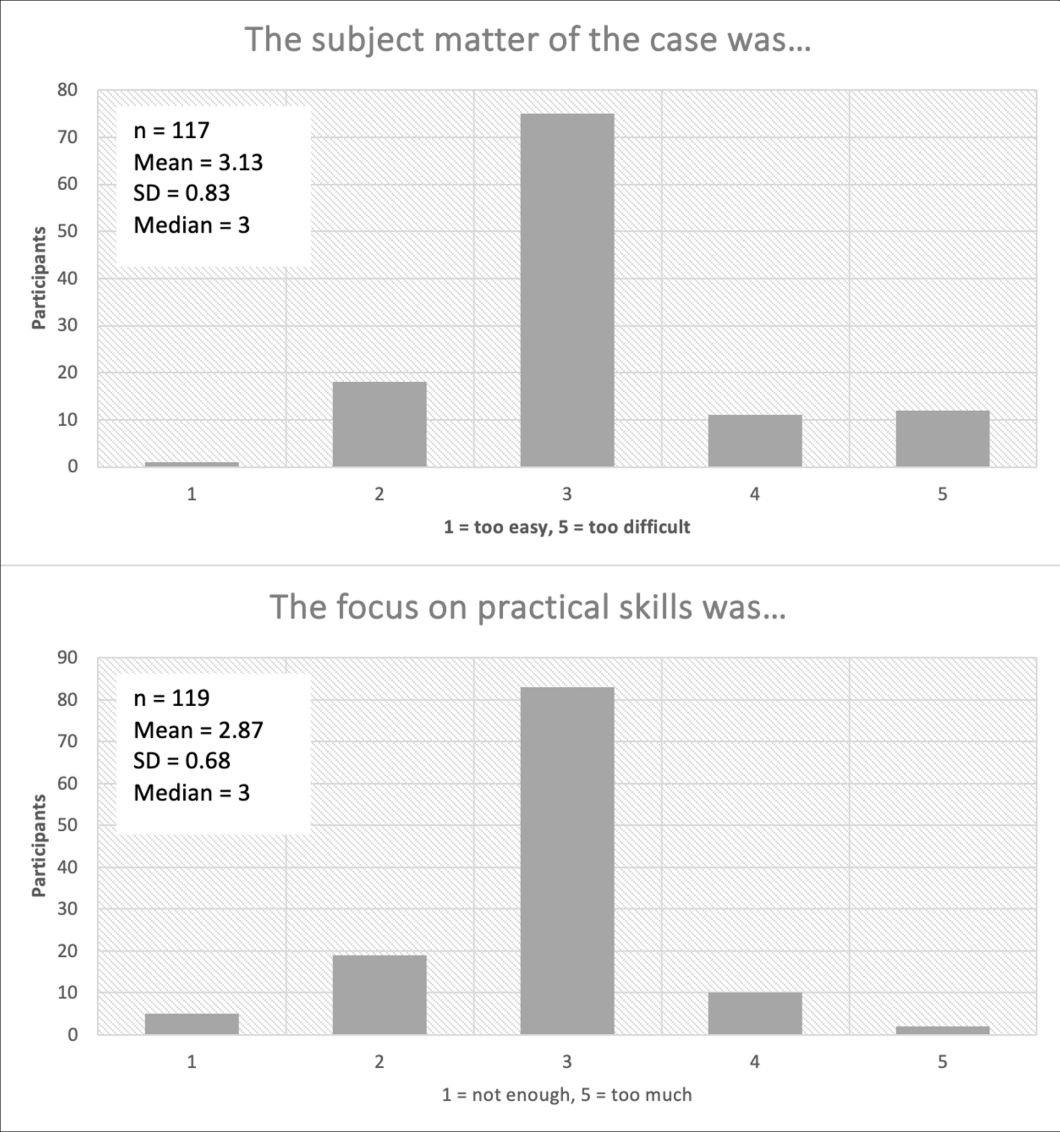
Evaluation of the Ready for Duty course, taking an excerpt from the survey of participants on the simulation of acute chest pain as an example. The evaluation was carried out directly at the end of each course day using a Likert ordinal scale from 1 to 5 (definition of the range shown under the x-axis of each diagram) on the basis of a survey on paper. n=number of participants; SD=standard deviation

**Figure 4:**
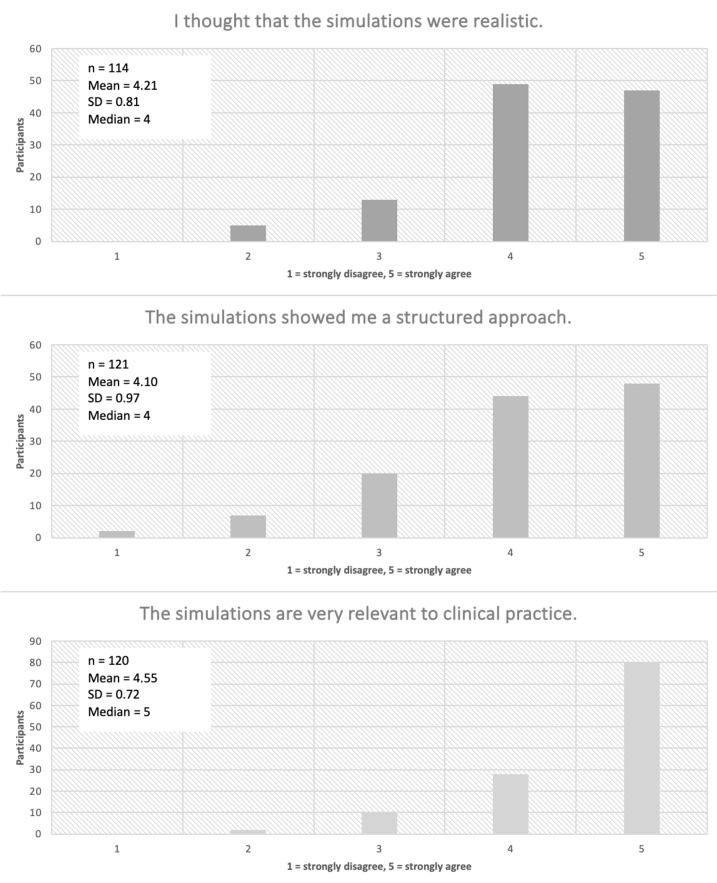
Evaluation of the ready for duty course showing an excerpt from the responses in the evaluation of the simulations. The evaluation was carried out directly at the end of each course day using a Likert ordinal scale from 1 to 5 (definition of the range shown under the x-axis of each diagram) on the basis of a survey on paper. n=number of participants; SD=standard deviation.

The curriculum has been taught 12 times to date; each time, at the end of each course day, participants were given the option of evaluating the course by completing a questionnaire created using evasys (Electric Paper Evaluation Systems GmbH, Lüneburg, Germany) and provided as a printout. In addition, after 3-6 months, they were sent an email inviting them to take part in an online survey on the long-term learning impact. In the questionnaires, statements are primarily rated using a five-point Likert scale (see figure 3 (Fig. 3) and figure 4 (Fig. 4) and attachment 1 for examples). In the following evaluation, scores of 4 and 5 on the ordinal scale from 1=“strongly disagree” to 5=“strongly agree” were considered as agreement.

**Figure 5:**
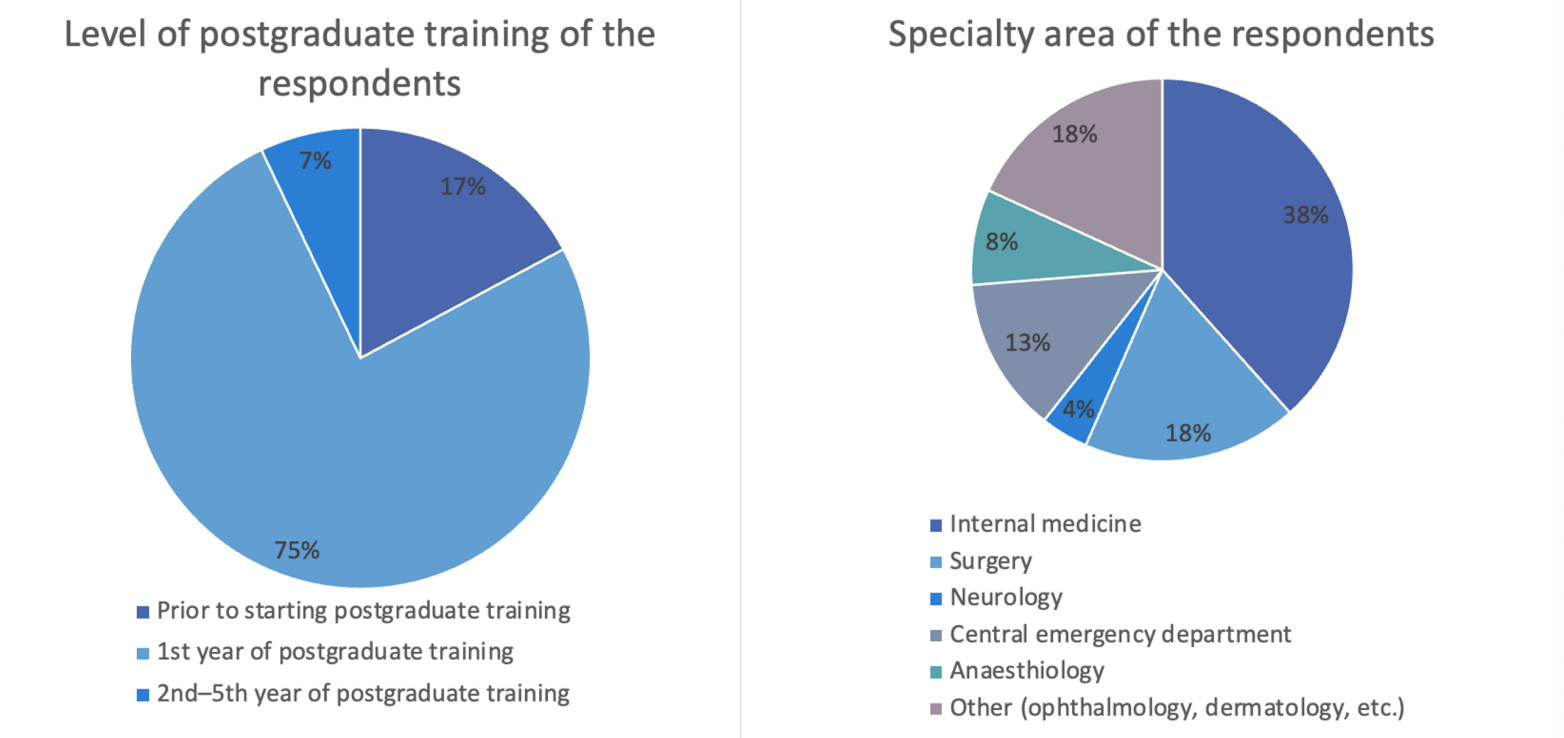
Level of postgraduate medical training and specialty area of the participants (n=120) of the evaluation of the ready for duty course

Since 2020, a total of 205 doctors have taken part in the Ready for Duty course, which has been held 12 times so far. Of these participants, 120 (58%) completed the evaluations (see figure 5 (Fig. 5)). 95% of the participants considered their newly acquired knowledge and skills to be useful in their subsequent clinical work. Participants rated simulations, a core element of the ready for duty course, as positive in terms of the learning impact (see figure 3 (Fig. 3)); 84% of the respondents said that they were realistic, and 76% rated working with the simulated patients as realistic.

The response rate for the long-term survey 3-6 months after completion of the course is currently 21%. Of the respondents, 95% said that, in hindsight, the course had enabled them to improve their practical skills, while 85% of the participants were able to integrate and apply these practical skills in their professional practice. 73% of the participants confirmed that they had improved their communication skills in the course, and 76% of the doctors were able to integrate these communication skills into their professional practice. In the long-term follow-up survey, 85% of the participants who were surveyed directly agreed that the course was useful for networking with colleagues within the hospital or at other hospitals.

Changes were made to the course based on the evaluation findings and interviews with the participants. For example, the station on inserting a urinary catheter was replaced by one on evaluating ECGs. Two elements were added to the communication units: one on communication with relatives in escalation situations and one using DocCom module on talking about dying. In addition, following experience during the pandemic, people had asked for training on how to communicate difficult information over the telephone, which was incorporated into the course. In future, as further courses are introduced, the course content will be modified on an ongoing basis to avoid redundancies. This will also support detailed preparation for each training section.

## 4. Discussion

It is difficult to objectively assess the learning impact among participants of the HaKom and the Ready for Duty course and the influence of the courses on the quality of patient care. A survey by questionnaire ultimately reflects the subjective view of the respondents. The response rate for the long-term follow-up survey on the Ready for Duty course is also low. Of those people who completed the questionnaire after 3-6 months, the majority confirmed that their practical and communication skills had improved and that this was relevant to their daily work. The aim of the course is to help participants care for patients confidently and competently. It is hoped that this will result in less stress for the doctors, will reduce the strain on hospitals and will also improve patient care.

The course is designed to provide an opportunity for effective learning and for applying medical knowledge in a safe environment [8], largely by using simulations; as already observed among students, it therefore offers an effective way to teach participants [9], [17], [18], supplementing online medical training. Simulations have the potential to increase participants’ confidence and clinical competence [19] and therefore play a key role in the other HaKom courses too.

In addition to the opportunity for simulation-based learning, the ready for duty course also offers participants a chance to practise their practical skills on models or on simulated patients. This can also help them learn the relevant entrustable professional activities (EPAs) as defined medical skills up to a point at which they can perform these activities autonomously themselves [20]. As a result of the opportunity to revise and develop practical and communication skills, doctors can become more confident in using these skills on patients [18], which may enable them to perform these tasks autonomously sooner.

In the long-term follow-up survey, 85% of the respondents confirmed that the course was useful for networking with colleagues within the hospital or at other hospitals. The HaKom courses may thus promote professional and interprofessional dialogue and networking within the region. It is hoped that networking between the participants can thus create a certain preference for the region.

## 5. Conclusion

As a firmly established part of the Hakom curriculum, the ready for duty course combines revision and learning of theory with the practical, site-specific application of this content for junior doctors at the beginning of their career. Extensive course content is taught, focusing on performing medical tasks in simulations and case-based learning. In summary, as part of the HaKom curriculum, the ready for duty course was rated as useful by participants from various different disciplines. On the basis of the evaluation findings, the programme has already been modified in the past using the ratings. Participants were able to enhance their practical and communication skills and increase their theoretical knowledge while revising structured procedures to treat specific conditions and practise them in depth in the safe environment of the simulations. This is designed to benefit the participants and ultimately to improve patient care. Increasing participants’ loyalty to the region might be another advantage for the hospitals. In future, the content of the courses that have already been established will be modified on the basis of the evaluation findings and by extending the range of courses on offer.

## Notes

### Contributions by the authors

BR, AS, DS and CL conducted the needs analysis. JS, AS, BR, JB, JA, LH and DS were involved in the initial design of the overall curriculum in general and specifically the Ready for Duty course. LH, SJ, LB and MS conducted interim evaluations and modified the curriculum accordingly. SJ, LB, LH, JA, MS, BR, AS, JS and DS play or have played a key role in running the Ready for Duty course four times a year. SJ, LB, LH and DS were responsible for producing the manuscript and the statistics, while all the authors were involved in revising and correcting the manuscript.

### Authors’ ORCIDs

- Susanna Jaspers: [0009-0003-0960-664X]
- Lena Bauer: [0009-0001-3526-938X]
- Linn Hempel: [0009-0009-5421-2029]
- Josefin Bosch: [0009-0005-1962-2293]
- Christiane Ludwig: [0009-0002-3724-954X]
- Ben Reufsteck: [0000-0003-0068-4820]
- Jonas Steglich: [0000-0002-5550-1481]
- Dietrich Stoevesandt: [0000-0001-5105-4488]

### Provision of data

The data on which the results are based can be obtained on request from the corresponding author.

## Competing interests

The authors declare that they have no competing interests. 

## Supplementary Material

Evaluation of the ready for duty course as an example of the follow-up survey 3-6 months after the course
