# Supplementary material for: HaKom – the Halle continuum as a postgraduate medical training curriculum, illustrated using the development and delivery of the ready for duty course
Source: GMS J Med Educ. 2024 Nov 15;41(5):Doc59. doi: 10.3205/zma001714 (PMC11656174; doi:10.3205/zma001714)
Supplement: Evaluation of the ready for duty course as an example of the follow-up survey 3-6 months after the course [file JME-41-59-s-001.pdf]

**Attachment 1: Evaluation of the ready for duty course as an example of the follow-up survey 3-6 months after the course**

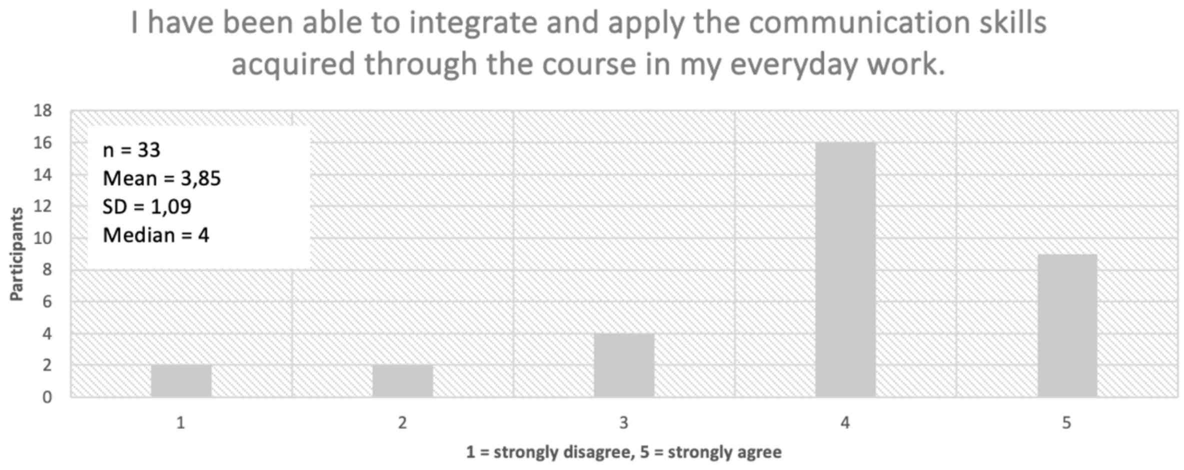


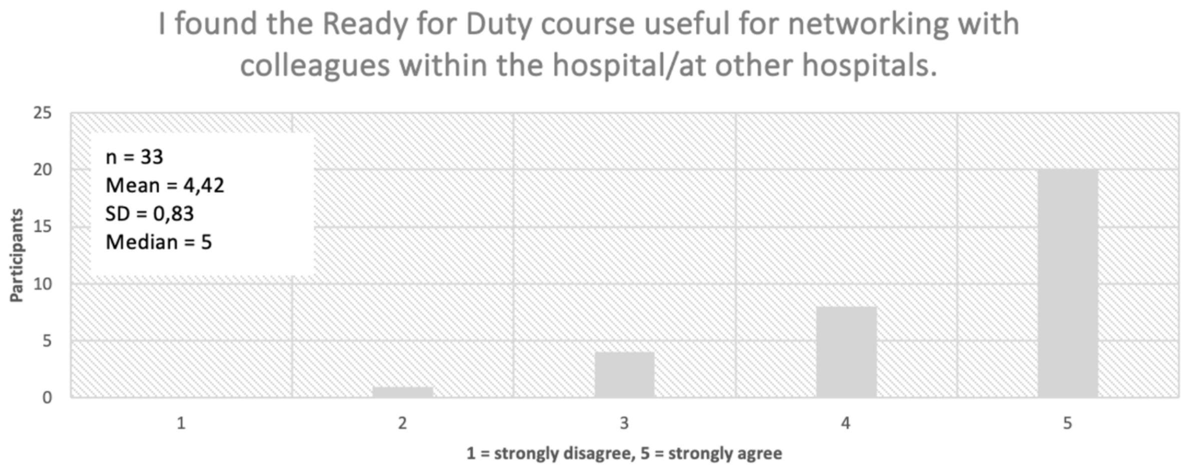


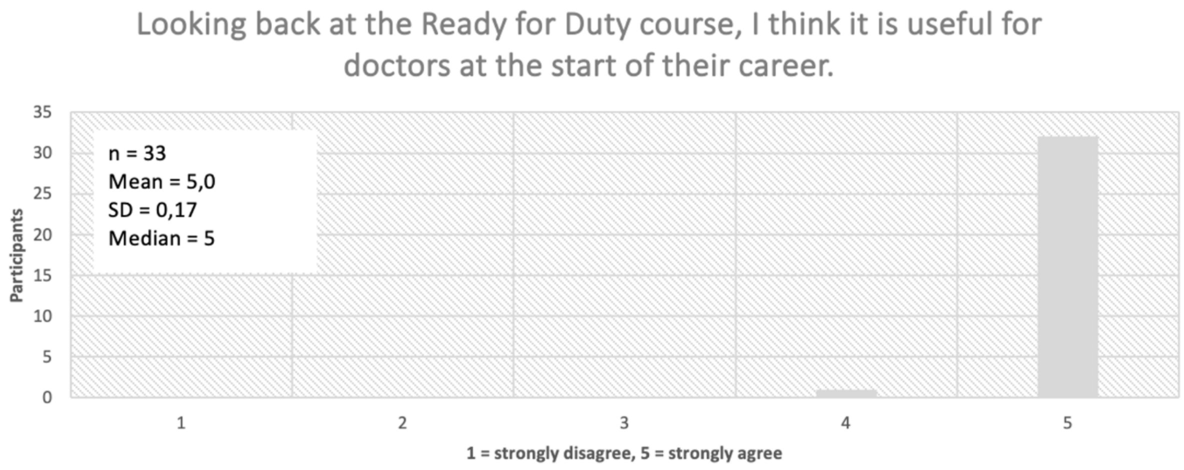


Attachment 1 to Jaspers S, Bauer L, Hempel L, Achenbach J, Bosch J, Ludwig C, Schwardt M, Reufsteck B, Siol A, Steglich J, Stoevesandt D. *HaKom – the Halle Continuum as a postgraduate medical training curriculum, illustrated using the development and delivery of the Ready for Duty course.* GMS J Med Educ. 2024;41(5):Doc59. DOI: 10.3205/zma001714

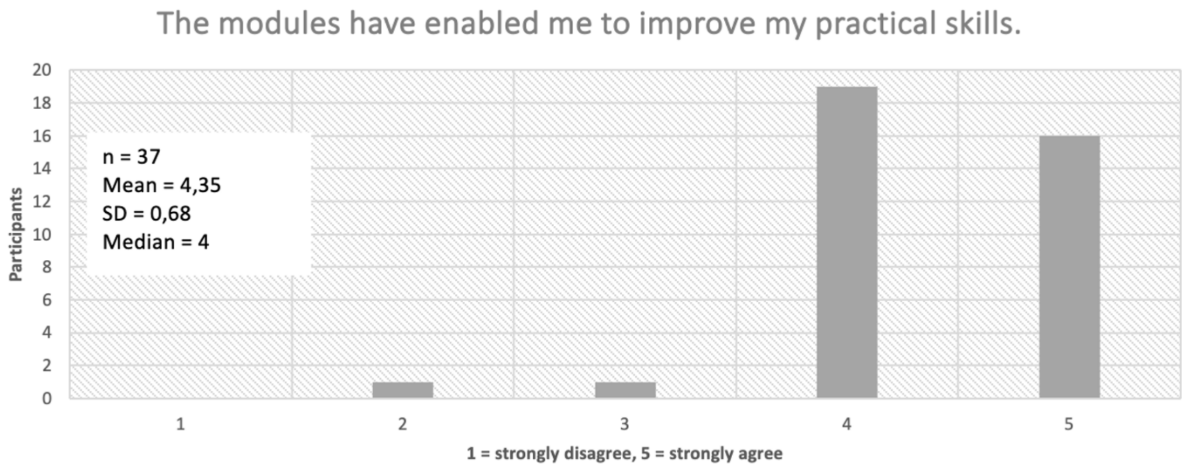


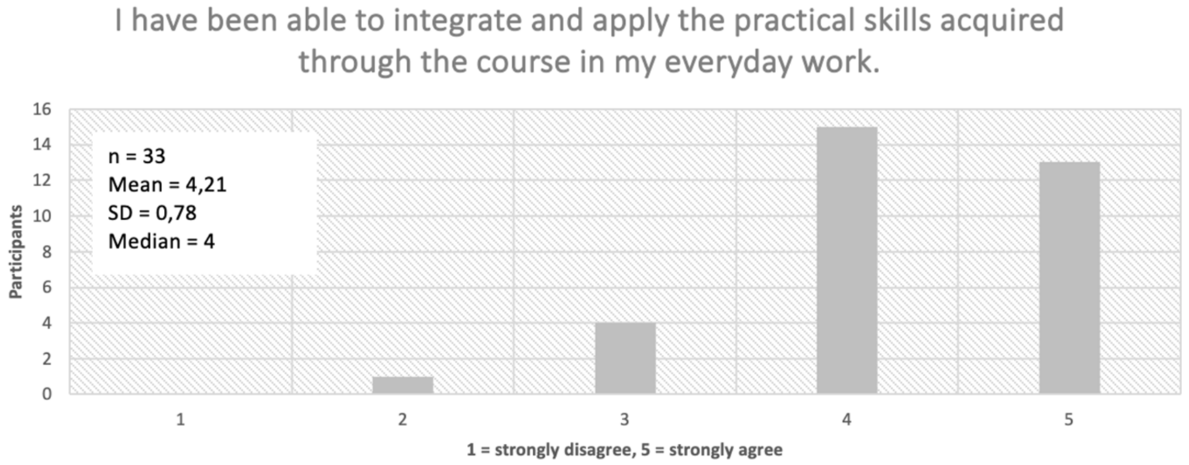


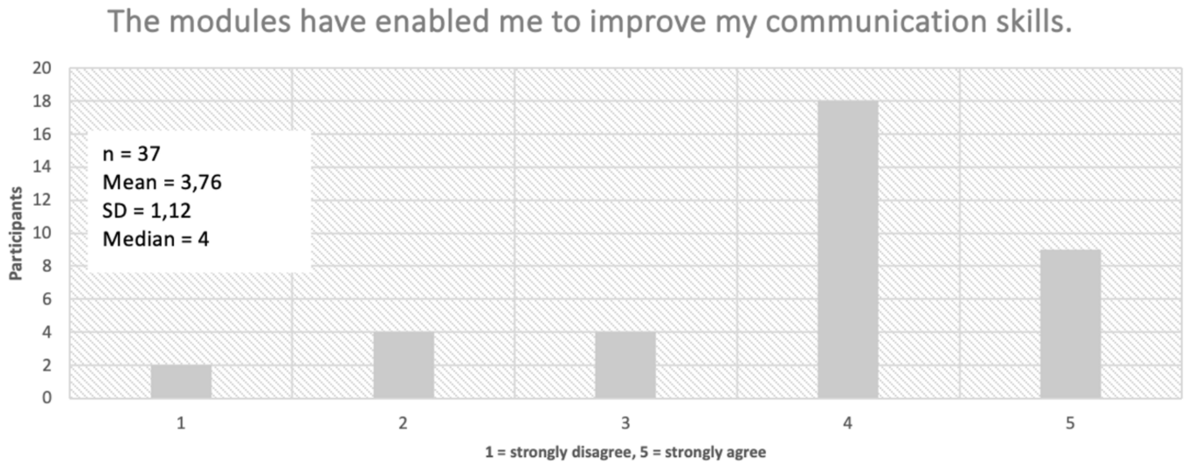


The evaluation was carried out using a Likert ordinal scale from 1 to 5 (definition of the range shown under the x-axis of each diagram) on the basis of a survey on paper. n=number of participants; SD=standard deviation.
